# Evaluation the immediate effects of bracing on kinetic parameters in adolescent idiopathic scoliosis patients

**DOI:** 10.1186/1748-7161-9-S1-O58

**Published:** 2014-12-04

**Authors:** Mohammad Taghi Karimi, Mahsa Kavyani, Mohammad Reza Etemadifar

**Affiliations:** 1 Musculoskeletal Research Center, Isfahan University of Medical Sciences, Isfahan, Iran; 2 Orthopedic Surgery Department, School of Medicine, Isfahan University of Medical Sciences, Isfahan, Iran

**Keywords:** Idiopathic scoliosis, brace, ground reaction force, stability

## Background

Idiopathic scoliosis induces change in coordination between body segments, spinal anatomy, left-right trunk symmetry and gait pattern. Various treatment methods have being used for scoliosis which includes: physical therapy, occupational therapy, osteopathic therapy, casting, bracing and surgery. However, using brace is a commonly used method in this regard. Although, the influence of brace to reduce the scoliosis curve has been investigated in lots of research studies, there is not enough research regarding the influence of brace on performance of scoliotic subjects while walking and standing. Therefore, the purpose of this study was to evaluate the immediate effect of brace on stability performance of scoliotic subjects and the symmetry of the ground reaction force applied on the right and left feet while walking.

## Method

Then girls aged between 8 and 12 years were recruited in this study. The gait analysis was assessed using a three-dimensional motion analysis and a force plate (Kistler) in two conditions, with and without Boston brace. Moreover their stability was evaluated by use of force plate. The difference of kinetic and stability parameters between two conditions(with and without Boston brace)was checked by use of paired T-test.

## Results

For scoliotic patients, comparison of in-brace and out-brace situations revealed a significant decrease in postural sway in brace associated with increase of patient stability. But very short-term bracing in AIS has no significant effect on the symmetry of force applied on right and left limbs during walking (p-value<0.05).

## Conclusion

Bracing aligned the vertebral column and improved the abilities of the subject to stand and walk.

